# A national population-based study of patients, bystanders and contextual factors associated with resuscitation in witnessed cardiac arrest: insight from the french RéAC registry

**DOI:** 10.1186/s12889-021-12269-4

**Published:** 2021-12-02

**Authors:** Paul-Georges Reuter, Valentine Baert, Hélène Colineaux, Joséphine Escutnaire, Nicolas Javaud, Cyrille Delpierre, Frédéric Adnet, Thomas Loeb, Sandrine Charpentier, Frédéric Lapostolle, Hervé Hubert, Sébastien Lamy

**Affiliations:** 1 Emergency Department, Toulouse University Hospital, 31000 Toulouse, France; 2 UMR 1027, Paul Sabatier University Toulouse III, Inserm, Toulouse, France; 3 AP-HP, SAMU 92, Hôpital Raymond Poincaré, 104, Boulevard Raymond Poincaré , 92380 Garches, France; 4 Évaluation des technologies de santé et des pratiques médicales, Univ. Lille, CHU Lille, 2694, F-59000 Lille, ULR France; 5 French National Out-of-Hospital Cardiac Arrest Registry, RéAC, Lille, France; 6 AP-HP, Urgences, Centre de Référence sur les Angioedèmes à Kinines, Hôpital Louis Mourier, Université de Paris, 92700 Colombes, France; 7 UF Recherche-Enseignement-Qualité, hôpital Avicenne, AP-HP, Université Paris, Urgences - Samu 93, 13, Inserm U942, 93000 Bobigny, France; 8 Group for Research and Analysis in Population Health (GAP), Claudius Regaud Institute, IUCT-O, Toulouse, France

**Keywords:** Out-of-hospital cardiac arrest, Deprivation, Bystander, Cardiopulmonary resuscitation, Registry

## Abstract

**Background:**

In out-of-hospital cardiac arrest (OHCA), bystander initiated cardiopulmonary resuscitation (CPR) increases the chance of return of spontaneous circulation and survival with a favourable neurological status. Socioeconomic disparities have been highlighted in OHCA field. In areas with the lowest average socioeconomic status, OHCA incidence increased, and bystander CPR decreased. Evaluations were performed on restricted geographical area, and European evaluation is lacking. We aimed to analyse, at a national level, the impact of area-level social deprivation on the initiation of CPR in case of a witnessed OHCA.

**Methods:**

We included all witnessed OHCA cases with age over 18 years from July 2011 to July 2018 form the OHCA French national registry. We excluded OHCA occurred in front of rescue teams or in nursing home, and patients with incomplete address or partial geocoding. We collected data from context, bystander and patient. The area-level social deprivation was estimated by the French version of the European Deprivation Index (in quintile) associated with the place where OHCA occurred. We assessed the associations between Utstein variables and social deprivation level using a mixed-effect logit model with bystander-initiated CPR.

**Results:**

We included 23,979 witnessed OHCA of which 12,299 (51%) had a bystander-initiated CPR. More than one third of the OHCA (8,326 (35%)) occurred in an area from the highest quintile of social deprivation. The higher the area-level deprivation, the less the proportion of bystander-initiated CPR (56% in Quintile 1 versus 48% in Quintile 5). The In the multivariable analysis, bystander less often began CPR in areas with the highest deprivation level, compared to those with the lowest deprivation level (OR=0.69, IC95%: 0.63-0.75).

**Conclusions:**

The level of social deprivation of the area where OHCA occurred was associated with bystander-initiated CPR. It decreased in the more deprived areas although these areas also concentrate more younger patients.

**Supplementary Information:**

The online version contains supplementary material available at 10.1186/s12889-021-12269-4.

## Background

Out-of-hospital cardiac arrests (OHCA) survival rates depend on numerous factors and oscillate, in Europe, between 3% and 10% [1]. Even if OHCA is a major public health concern in most of industrialised countries, these rates have not substantially improved these 20 past years, leading to approximately 46,000 deaths per year in France [2].

Victims’ medical history and behaviours such as history of cardiovascular diseases, diabetes, obesity, alcoholism, and tobacco use are known factors of survival chances decrease. However, some studies have revealed an improvement of OHCA survival rates in some contexts. Hence, the Swedish study of Strömsöe et al. led in 2015 revealed that survival rates doubled between 1992 and 2011 [3]. Lai et al. observed the same tendency between 2001 and 2012 in Singapore [4]. The reinforcement of the first links of the chain of survival seems to be a main factor of success [3, 4]. These first links do not depend on professional mobile teams but rely on the ability of bystanders to recognise an OHCA and provide adequate care [5, 6]. Bystander-initiated cardiopulmonary resuscitation (CPR) rates remain low in many countries despite information campaigns and institutional encouragements [2, 7, 8]. Depending on the country, this percentage varied from 13 to 83%, although that includes all witness statuses [9]. When initiated, a bystander CPR increases the chance of return of spontaneous circulation (ROSC). It also strongly increases 30-days survival and a favourable neurological recovery at discharge [10, 11]. It is known that OHCA onset incidence is often subject to territorial and socioeconomic disparities with higher incidence rates in areas with the lowest average socioeconomic status (SES) [12, 13].

A recent meta-analysis synthesized the impact of deprivation on the incidence, bystander CPR and survival in case of OHCA [14]. Deprivation was analysed on the basis of three levels: the individuals, the area of OHCA victims residence, and the area where the OHCA occurred. The impact on the bystander CPR was assessed using the average SES level of the area where OHCA occurred [14]. They suggested that OHCA occurring in deprived areas were less likely to benefit from bystander-initiated CPR. On the 13 studies analysed in this meta-analysis, only two were European and took place in United Kingdom [15, 16]. To our knowledge, no study was conducted at a national level. Another European study dealing with this topic and not included in the meta-analysis was carried out by Dahan et al. and focused only on the city of Paris [17]. In this context, we propose to assess, based on data from a national registry, if bystander CPR is differently initiated according to the social deprivation level of the OHCA onset area.

## Methods

### Prehospital emergency medical system (EMS)

In France, the pre-hospital emergency medical system (EMS) is a two-tier, physician-based system. The medical dispatch centre sends professional emergency personnel, including firemen (typically the first responders that provides basic cardiac life support (BCLS)) and/or mobile intensive care units (MICU), i.e. an ambulance with an emergency physician that implements advanced cardiac life support (ACLS) [18].

### Study setting

Study data were gathered in the French national OHCA register (RéAC) [2]. All participating medical emergency response systems (MERS) use a specific RéAC form to fill in context, care, times and immediate vital status of OHCA victims. This form meets the requirement of the Utstein universal style [19]. If patients are alive at hospital admission, a follow-up at 30 days after OHCA or at hospital discharge is completed. Some quality controls are performed on the RéAC database (on- or off- line). A clinical research associate check randomly 10% of records. Furthermore, automatically controls are implemented to detect errors, inconsistencies or out-of-bound values. Currently, the RéAC covered about 20% of the French population.

This medical registry assessment was approved by the French Advisory Committee on Information Processing in Health Research (CCTIRS) and by the French National Data Protection Commission (CNIL, authorisation number 910,946). This study was approved as a medical assessment registry without the requirement for patient consent.

We selected all witnessed OHCA cases aged over 18 years, from July 2011 to July 2018. Exclusion criteria were age under 18 years, OHCA in front of rescue teams (BCLS or ACLS teams), no witness, incomplete address or geocoding, and OHCA occurred in nursing homes.

### Data collection

Three types of data were collected: (i) context-related data including the address and its location (home, public place or other), and the timing: weekday, school vacation periods, time of the call (day from 08:00 to 19:59 or night from 20:00 to 07:59) and if it occurred during working hours (day time from Monday to Friday except public holiday); (ii) bystander-related data including the type (family, health professional, rescuer or others), if telephonic CPR (tCPR) was suggested, and if CPR was initiated; (iii) patient-related data including gender, age, the causes of OHCA (described with Ustein style), and previous known cardiovascular diseases. Because of a high rate of missing data regarding the tCPR, this variable was categorised in “Yes”, “No” and “Missing value”.

### Primary endpoint and variables of interests

The primary endpoint was the initiation of bystander CPR. The main variable of interest was the social deprivation level of the OHCA onset area. Social deprivation was defined as a state of observable and demonstrable disadvantage relative to the local community or the wider society to which an individual, family or group belongs, according to Townsend et al. [20]. We used an ecological measure of deprivation, the French version of the European deprivation Index (EDI) [21]. It is an aggregated (ecological) score of relative deprivation which can be calculated for each European country and for various levels of granularity (i.e.: counties, cities, neighbourhood) using an European survey specifically devoted to assessed relative deprivation and national census data. For each country, the national version of EDI is a weighted combination of census-aggregated variables that are most highly correlated with country-specific individual deprivation indicator. For now EDI is developed for France, Italy, England, Slovenia, Portugal and Spain [22–24]. We computed EDI at the IRIS (Ilôts Regroupés Pour l’Information Statistique) level, which corresponds to a small neighbourhood with homogeneous socio-demographical data. The French version of EDI assesses the following dimensions: overcrowded housing, access to heating, non-owners, unemployment, foreign nationality inhabitants, access to a car, unskilled/agricultural workers, households with at least 6 people, inhabitants’ level of education, the number of single-parent families. We obtained IRIS by geocoding the OHCA addresses. Then, the EDI score was computed for each IRIS and categorised into quintile ranging from 1 (the least deprived) to 5 (the most deprived). We used QGIS (Development Team, 2009. QGIS Geographic Information System. Open Source Geospatial Foundation. URL http://qgis.org) for geocoding. The co-variables of interest were other variables described above.

### Statistical analysis

The results of this study are reported in accordance with the STROBE checklist for observational studies.

Data were expressed as numbers with percentages for categorical variables or means with standard deviation or medians with interquartile range [IQR] for continuous variables. Categorical data were compared by chi-square or Fisher exact test when appropriate and continuous data by Student t or Mann and Whitney test as appropriate.

To explore the impact of the deprivation level on the primary outcome (bystander CPR), considering potential geographical variations (defined as French regions), we set up mixed-effect models. After verifying the association between EDI and the primary outcome, bivariate analyses were performed between co-variables and EDI and between co-variables and the primary outcome. Variables retained for the multivariable model were those associated with EDI and with the primary outcome at the threshold of 0.2. The models were adjusted for context, bystander, and patient data), separately first, then simultaneously.

All analyses were two-sided, and a p-value was considered as statistically significant for p < 0.05. Statistical analysis was performed using R software (version 3.6.0).

## Results

**Fig. 1:**
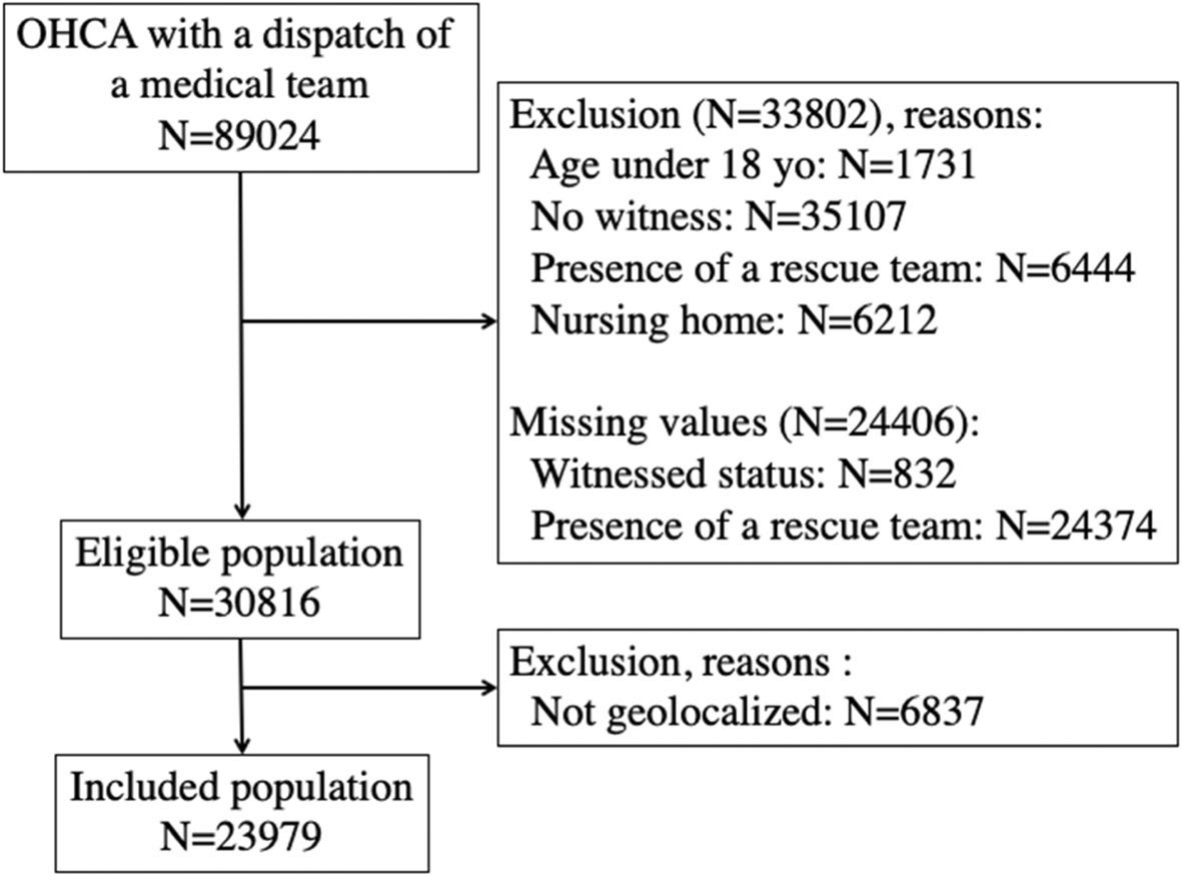
Flow chart. Legend: OHCA: Out-of-hospital cardiac arrest. The total of exclusions does not equal 100% because a patient may be excluded for several reasons

During the period study, 89,024 OHCA were recorded; 30,816 (35%) occurred for unwitnessed patient aged over 18 years old, without rescue team and outside a nursing home. Geocoding was impossible or partial for 6,837 patients due to missing or incomplete addresses, resulting in 23,979 OHCA included (Fig. 1). The sensitivity analysis regarding the excluded or included status is presented in the Additional file 1: Appendix 1.

The Table 1 presents the baseline characteristics of witnessed OHCA, proving both marginal and condition distribution by the deprivation level of the area where OHCA occurred. The highest level of deprivation population (EDI quintile 5) comprised 8,324 (35%) patients. In the highest deprivation level group, a tCPR was performed in 23% of patients (versus 26–28% in the other EDI groups). Patients from the most deprived group were younger and more often women than patients from the other group of deprivation. Co-variables associated with EDI were location, working hours, type of bystander, cause of OHCA and a known cardiovascular disease. Information regarding OHCA process and patient outcomes, in accordance with the Utstein style, are presented in the Additional file 1: Appendix 2.

**Table.1:** Bivariate analysis between the deprivation level and the co variables

	Total *N* = 23979	EDI					*p* value*
		1 (the least deprived) *N*=4055	2 *N*=3784	3 *N*=3553	4 *N*=4263	5 (the most deprived) *N*=8324	
Context data							
Location							0.006
At home	17683 (74.6)	3059 (76.2)	2780 (74.4)	2620 (74.6)	3143 (74.5)	6081 (73.9)	
Public place	5356 (22.6)	859 (21.4)	857 (22.9)	788 (22.4)	935 (22.2)	1917 (23.3)	
Other	676 (2.9)	99 (2.5)	100 (2.7)	104 (3.0)	138 (3.3)	235 (2.9)	
Public holiday							0.910
No	23168 (96.6)	3919 (96.6)	3674 (97.1)	3427 (96.5)	4122 (96.7)	8026 (96.4)	
Yes	811 (3.4)	136 (3.4)	110 (2.9)	126 (3.5)	141 (3.3)	298 (3.6)	
Time of the call							0.203
Day (0800-1959)	15281 (63.9)	2630 (64.9)	2414 (63.8)	2260 (63.6)	2748 (64.6)	5229 (63.1)	
Night (2000-0759)	8647 (36.1)	1421 (35.1)	1367 (36.2)	1291 (36.4)	1506 (35.4)	3062 (36.9)	
Working hours							0.176
No	13629 (57.0)	2263 (55.9)	2182 (57.7)	2069 (58.3)	2391 (56.2)	4724 (57.0)	
Yes	10299 (43.0)	1788 (44.1)	1599 (42.3)	1482 (41.7)	1863 (43.8)	3567 (43.0)	
Bystander data							
Type of bystander							0.205
Family	15838 (66.1)	2714 (67.0)	2487 (65.8)	2332 (65.8)	2796 (65.7)	5509 (66.2)	
Health prof.	2602 (10.9)	447 (11.0)	447 (11.8)	393 (11.1)	469 (11.0)	846 (10.2)	
Rescuer	888 (3.7)	143 (3.5)	150 (4.0)	144 (4.1)	170 (4.0)	281 (3.4)	
Other	4617 (19.3)	746 (18.4)	695 (18.4)	677 (19.1)	819 (19.3)	1680 (20.2)	
tCPR							**0.003**
Yes	6070 (25.3)	1152 (28.4)	964 (25.5)	941 (26.5)	1113 (26.1)	1900 (22.8)	
No	7328 (30.6)	1251 (30.9)	1163 (30.7)	1092 (30.7)	1318 (30.9)	2504 (30.1)	
Missing value	10581 (44.1)	1652 (40.7)	1657 (43.8)	1520 (42.8)	1832 (43.0)	3920 (47.1)	
Patient data							
Gender							**<0.001**
Female	7509 (31.3)	1129 (37.8)	1171 (30.9)	1079 (30.4)	1361 (31.9)	2769 (33.3)	
Male	16466 (68.7)	2925 (72.2)	2613 (69.1)	2473 (69.6)	2901 (68.1)	5554 (66.7)	
Age, by quartile							**0.003**
[18,56]	6157 (25.7)	946 (23.3)	917 (24.2)	867 (24.4)	1086 (25.5)	2341 (28.1)	
(56,69]	6044 (25.2)	1048 (25.8)	938 (24.8)	905 (25.5)	1040 (24.4)	2113 (25.4)	
(69,81]	6209 (25.9)	1084 (26.7)	1032 (27.3)	923 (26.0)	1081 (25.4)	2089 (25.1)	
(81,108]	5569 (23.2)	977 (24.1)	897 (23.7)	858 (24.1)	1056 (24.8)	1781 (21.4)	
Cause of the CA							**0.029**
Med. cardiac	15751 (65.7)	2651 (65.4)	2441 (64.5)	2370 (66.7)	2800 (65.7)	5489 (65.9)	
Med. non cardiac	4615 (19.2)	784 (19.3)	740 (19.6)	667 (18.8)	821 (19.3)	1603 (19.3)	
Asphyxia	1200 (5.0)	176 (4.3)	204 (5.4)	187 (5.3)	218 (5.1)	415 (5.0)	
Traumatic	1852 (7.7)	338 (8.3)	304 (8.0)	259 (7.3)	324 (7.6)	627 (7.5)	
Drowning	390 (1.6)	82 (2.0)	73 (1.9)	52 (1.5)	68 (1.6)	115 (1.4)	
Intox./Drug overdose	160 (0.7)	24 (0.6)	20 (0.5)	18 (0.5)	26 (0.6)	72 (0.9)	
Electrocution	11 (0)	0 (0)	2 (0.1)	0 (0)	6 (0.1)	3 (0.0)	
Cardiovasc. disease							0. 151
Unknown	10540 (44.0)	2257 (55.7)	2130 (56.3)	2035 (57.3)	2364 (55.5)	4653 (55.9)	
Yes	13439 (56.0)	1798 (44.3)	1654 (43.7)	1518 (42.7)	1899 (44.5)	3671 (44.1)	

Categorical variables are presented with number and percentage

CA: Cardiac arrest, CPR: Cardiopulmonary resuscitation, tCPR: CPR assisted by telephone, EDI: European Deprivation Index

*p-values from the log-likelihood test comparing nested mixed effect models with and without EDI as explanatory variable

The Table 2 presents the bivariate analysis between the bystander-initiated CPR and EDI or the co-variables. The deprivation level associated with the area where OHCA occurred was associated with bystander CPR initiation. The higher the deprivation, the less the bystander CPR initiation, representing 2258/4055 (56%) (EDI quintile 1) versus 3951/8324 (48%) (EDI quintile 5). A CPR was more often initiated when OHCA occurred outside the home (30% versus 20%), during working hours (46% versus 40%), by a bystander other than family (44% versus 23%), when a tCPR was performed (40% versus 10%) and on younger patients. Other co-variables associated with the likelihood of occurrence of a bystander CPR were time of the call, patient’s gender, cause of the OHCA and a known cardiovascular disease.

**Table.2:** Bivariate analysis between the bystander-initiated CPR and EDI or the co-variables

	Initiated CPR *N*= 12299	No initiated CPR *N*= 11680	OR (IC 95%)	*p* value
EDI (quintile)				
1 – the least deprived	2258 (18.4)	1797 (15.4)	1	
2	2040 (16.6)	1744 (14.9)	0.93 (0.85-1.01)	0.098
3	1891 (15.4)	1662 (14.2)	0.92 (0.84-1.01)	0.075
4	2159 (17.6)	2104 (18.0)	0.85 (0.78-0.93)	**<0.001**
5 - the most deprived	3951 (32.1)	4373 (37.4)	0.76 (0.70-0.82)	**<0.001**
Context data				
Location				
At home	8492 (69.8)	9191 (79.6)	Ref	
Public place	3217 (26.4)	2139 (18.5)	1.61 (1.51-1.71)	**<0.001**
Other	457 (3.8)	219 (1.9)	2.26 (1.92-2.67)	**<0.001**
Public holiday				0.592
No	11892 (96.7)	11276 (96.5)	Ref	
Yes	407 (3.3)	404 (3.5)	0.96 (0.84-1.11)	
Time of the call				**<0.001**
Day (0800-1959)	8364 (68.1)	6917 (59.4)	Ref	
Night (2000-0759)	3914 (31.9)	4733 (40.6)	0.68 (0.65-0.72)	
Working hours				**<0.001**
No	6627 (54.0)	7002 (60.1)	Ref	
Yes	5651 (46.0)	4648 (39.9)	1.28 (1.22-1.35)	
Bystander data				
Type of bystander				
Family	6903 (56.2)	8935 (76.7)	Ref	
Health prof.	2183 (17.8)	419 (3.6)	6.78 (6.08-7.56)	**<0.001**
Rescuer	799 (6.5)	89 (0.8)	11.53 (9.23-14.40)	**<0.001**
Other	2406 (19.6)	2211 (19.0)	1.40 (1.31-1.50)	**<0.001**
tCPR				
Yes	4926 (40.1)	1144 (9.8)	Ref	
No	2364 (19.2)	4966 (42.5)	0.11 (0.10-0.12)	**<0.001**
Missing value	5009 (40.7)	5576 (47.7)	0.21 (0.19-0.22)	**<0.001**
Patient data				
Gender				**<0.001**
Female	3687 (30.0)	3822 (32.7)	Ref	
Male	8611 (70.0)	7855 (67.3)	1.13 (1.07-1.19)	
Age, by quartile				
[18,56]	3575 (29.1)	2582 (22.1)	Ref	
(56,69]	3336 (27.1)	2708 (23.2)	0.89 (0.83-0.96)	**0.002**
(69,81]	3000 (24.4)	3209 (27.5)	0.67 (0.62-0.72)	**<0.001**
(81,108]	2388 (19.4)	3181 (27.2)	0.54 (0.51-0.59)	**<0.001**
Cause of the CA				
Med. cardiac	8545 (69.5)	7206 (61.7)	Ref	
Med. non cardiac	2122 (17.3)	2493 (21.3)	0.72 (0.68-0.77)	**<0.001**
Asphyxia	631 (5.1)	569 (4.9)	0.94 (0.83-1.06)	0.285
Traumatic	637 (5.2)	1215 (10.4)	0.44 (0.40-0.49)	**<0.001**
Drowning	268 (2.2)	122 (1.0)	1.81 (1.46-2.25)	**<0.001**
Intox./Drug overdose	88 (0.7)	72 (0.6)	1.04 (0.76-1.42)	0.827
Electrocution	8 (0.1)	3 (0.0)	2.29 (0.56-9.31)	0.247
Cardiovasc. disease				0.055
Unknown	6995 (56.9)	6444 (55.2)	Ref	
Yes	5304 (43.1)	5236 (44.8)	0.95 (0.90-1.00)	

Categorical variables are presented with number and percentage

CA: Cardiac arrest, CPR: Cardiopulmonary resuscitation, tCPR: CPR assisted by telephone, EDI: European Deprivation Index

The multivariable analysis is presented in the Table 3. Our results show that compared to the lowest level of deprivation (EDI quintile 1), bystander less often began CPR in the areas associated with the highest deprivation level (EDI quintile 5, OR [CI95%]=0.69[0.63; 0.75]). In addition, CPR initiations were less frequent in absence of performed tCPR, arising older age, and in the case of a non-cardiac or a traumatic cause of OHCA. In contrary, CPR initiations were more frequent where OHCA occurred out-of-home, in working hours, when the bystander was other than family, and in the case of a drowning cause.

**Table.3:** Multivariable model for initiation of bystander cardiopulmonary resuscitation

	OR (IC 95%)	*p* value
EDI (quintile)		
1 – the least deprived	Ref	
2	0.93 (0.84-1.04)	0.213
3	0.89 (0.80-0.99)	**0.032**
4	0.78 (0.71-0.87)	**<0.001**
5 - the most deprived	0.69 (0.63-0.75)	**<0.001**
Location		
At home	Ref	
Public place	1.72 (1.55-1.90)	**<0.001**
Other	1.61 (1.32-1.96)	**<0.001**
Working hours		**<0.001**
No	Ref	
Yes	1.11 (1.05-1.19)	
Type of bystander		
Family	Ref	
Health prof.	11.13 (9.87-12.56)	**<0.001**
Rescuer	17.21 (13.58-21.80)	**<0.001**
Other	1.41 (1.28-1.56)	**<0.001**
tCPR		
Yes	Ref	
No	0.08 (0.07-0.09)	**<0.001**
Missing value	0.16 (0.15-0.18)	**<0.001**
Gender		0.727
Female	Ref	
Male	1.01 (0.95-1.08)	
Age, by quartile		
[18,57]	Ref	
(57,70]	0.80 (0.73-0.87)	**<0.001**
(70,82]	0.62 (0.56-0.68)	**<0.001**
(82,108]	0.46 (0.41-0.50)	**<0.001**
Cause of the CA		
Med. cardiac	Ref	
Med. non cardiac	0.78 (0.72-0.84)	**<0.001**
Asphyxia	1.10 (0.95-1.26)	0.197
Traumatic	0.25 (0.22-0.29)	**<0.001**
Drowning	1.40 (1.09-1.80)	**0.008**
Intox./Drug overdose	0.82 (0.57-1.18)	0.763
Electrocution	1.25 (0.29-5.44)	0.264
Cardiovasc. disease		0.264
Unknown	Ref	
Yes	1.04 (0.97-1.11)	

The multivariable analysis was performed on 23,634 patients without missing data

CA: Cardiac arrest, CPR: Cardiopulmonary resuscitation, tCPR: CPR assisted by telephone, EDI: European Deprivation Index, OR: odds ratio

## Discussion

In the French national registry, we observed disparities in OHCA rate, patient’s age and initiation of bystander CPR according to the level of social deprivation of the place where OHCA occurred. These results persist after taking co-variables into account in multivariable analyses. This translates that the observed association between the social deprivation level of the place where a witnessed OHCA occurred and the CPR initiation by the bystander was not explained by differences in both the context, patients, and bystander characteristics included in the models.

In our study, we found more than one third of OHCA occurred in a high deprivation area. This over-representation of the most deprived areas in the OHCA caseload could support the impact of the area level of deprivation on OHCA incidence [13]. In the multivariable analysis, the OHCA area level social deprivation was negatively associated with the CPR initiation by the bystander. This observation is reinforced when considering that patients were younger in the most deprived areas. A challenge related to the optimised implementation of the chain of survival is the limitation of no-flow duration, that is bystanders’ ability to initiate CPR immediately after recognising OHCA [19]. Regardless the location of the OHCA, its aetiology or patient’s characteristics (e.g. gender, age), ROSC likelihood of occurrence and survival rate depend on the no-flow duration [25]. Regarding OHCA prognosis and outcome, patient’s age was described as negative predictor of overall survival and of survival with good neurological outcome [26, 27]. In our study, patients with OHCA in the area with the highest social deprivation level were younger and also with the fewest bystander-initiated CRP rate.

In the Korea nationwide registry, the temporal trend in bystander CPR increased in a different way considering the area average socioeconomic status. In ten years, the rate of bystander CPR increased by 22% in the least deprived areas versus 11% in the most deprived areas [28]. Public access to a defibrillator also varied widely depending on social factors. Lee et al. estimated that there were 12.7 defibrillators per 10,000 persons in the least deprived areas versus 4.9 defibrillators per 10,000 persons in the most deprived areas [29]. There are still considerable efforts to implement for improving the first link in the chain of survival. Until society becomes more aware, the use of mobile phone technology could bridge the current gap. Several software exists on smartphones that aim at helping to recruit laypersons to perform CPR while waiting the rescue teams [30]. Our findings point out the need to improve the bystander-initiated CPR rate in the most deprived area.

Our study has some limitations. We excluded numerous patients due to missing values or the impossibility to geocode the address, raising concerns about selection bias (Supplementary data, Table 1). However, results from Additional file 1: appendix 1 show differences between the included and excluded populations that are not clinically relevant although statistically significant, likely due to the width of the sample size. In addition, missing data in covariables included in the multivariable model concerned less 2% of the whole sample, minimizing the risk of selection bias. The sensitivity analyses carried on after the imputation of the missing data using a multiple imputation method provide similar results, supporting the robustness of our observations (Supplementary data, Table 2). As said above, we were not able to collect the bystanders’ individual socioeconomic position. We used a validated ecological index, the French EDI [31]. It was developed to allow comparison between European countries and used to approach the individual-level position in various studies [32–35]. The EDI value was assessed from the residence address of the inhabitants of the area where OHCA occurred. Thus, the association between bystander CPR and OHCA-area deprivation may reflect an environmental effect as well, due to the area intrinsic characteristics as the effect associated with the characteristics of the whole daily population (i.e. the population generally occupying the area), or both. The underlying mechanisms remain to be investigated using more specific tools allowing for assessing the time-dynamic variation of areas social composition, (for instance the Mobiliscope project, www.mobiliscope.cnrs.fr), as well as through a more qualitative approach to investigate the potential brakes and levers linked with CPR initiation by bystander. Finally, despite these limitations, we were able to provide a nationwide assessment of the association between the deprivation level of the area where OHCA occurred and CPR initiation by the bystander, supporting the idea of a need of improvement if the more deprived areas. This study constitutes the first steps for a future in depth analyse of the pathways linking the social deprivation of the area where OHCA occurred to bystander CPR initiation, through a causal perspective.

## Conclusions

Our study is the first study at a national scale investigating the impact of social deprivation level of the onset area on the bystander-initiation CPR. The deprivation level of the area where OHCA occurred was associated with the probability of bystander-initiated CPR. It was at its lowest level in the area with the highest level of deprivation where the patients are younger, and the OHCA incidence higher. These findings underline the need to investigate both the characteristics of the physical environment and the populations living or conducting daily activities in these areas. These analyses will provide a better understanding of the potential target to improve the chain of survival.

## Supplementary Information

**Additional file 1.**

**Additional file 2.**

## Data Availability

The datasets used and/or analysed during the current study are available from the corresponding author on reasonable request.

## Abbreviations

ACLS: advanced cardiac life support
AIC: Akaike information criterion
BCLS: basic cardiac life support
CPR: cardiopulmonary resuscitation
EDI: European deprivation Index
EMS: emergency medical system
MERS: medical emergency response systems
MICU: mobile intensive care units
OHCA: Out-of-hospital cardiac arrests
ROSC: return of spontaneous circulation
SES: socioeconomic status
tCPR: telephonic cardiopulmonary resuscitation
