# Supplementary material for: A national population-based study of patients, bystanders and contextual factors associated with resuscitation in witnessed cardiac arrest: insight from the french RéAC registry
Source: BMC Public Health. 2021 Dec 2;21:2202. doi: 10.1186/s12889-021-12269-4 (PMC8638114; doi:10.1186/s12889-021-12269-4)
Supplement: Supplementary file 1 — Additional file 1. [file 12889_2021_12269_MOESM1_ESM.docx]

**A national population-based study of patients, bystanders and contextual factors associated with resuscitation in witnessed cardiac arrest: insight from the French RéAC registry**

Paul-Georges Reuter, M.D.,Ph.D.^1,2,3^, Valentine Baert, Ph.D.^4,5^, Hélène Colineaux, M.D., M.S.^2^, Joséphine Escutnaire, Ph.D.^4^, Nicolas Javaud, M.D.,Ph.D.^6^, Cyrille Delpierre, Ph.D.^2^, Frédéric Adnet, M.D.,Ph.D.^7^, Thomas Loeb, M.D.^3^, Sandrine Charpentier, M.D.,Ph.D.^1,2^, Frédéric Lapostolle, M.D.,Ph.D.^7^, Hervé Hubert, Ph.D.^4,5^, Sébastien Lamy, Ph.D.^2, 8^

***Affiliations***

1 Emergency Department, Toulouse University Hospital, 31000, Toulouse, France.

2 UMR 1027, Paul Sabatier University Toulouse III, Inserm, Toulouse, France.

3 AP-HP, SAMU 92, Hôpital Raymond Poincaré, 92380 Garches, France.

4 Univ. Lille, CHU Lille, ULR 2694 - METRICS : Évaluation des technologies de santé et des pratiques médicales, F-59000 Lille, France

5 French National Out-of-Hospital Cardiac Arrest Registry, RéAC, Lille, France.

6 AP-HP, Urgences, Centre de référence sur les angioedèmes à kinines, hôpital Louis Mourier, Université de Paris, 92700 Colombes, France

7 AP-HP, Urgences - Samu 93, UF Recherche-Enseignement-Qualité, hôpital Avicenne, Université Paris 13, Inserm U942, 93000 Bobigny, France

8 Group for research and analysis in population health (GAP), Claudius Regaud Institute, IUCT-O, Toulouse, FRANCE

Appendix

Appendix 1. Baseline characteristics according to the included or excluded status – sensitivity analysis

|  | Included population  N = 23979 | Excluded population  N = 65045 | p value |
| --- | --- | --- | --- |
| Context data |  |  |  |
| Location  At home  Public place  Other  NA | 17683 (73.7)  5356 (22.3)  676 (2.8)  264 (1.1) | 43318 (66.6)  8801 (13.5)  1523 (2.3)  11403 (17.5) | **<0.0001** |
| Day  Monday  Tuesday  Wednesday  Thursday  Friday  Saturday  Sunday  NA | 3446 (14.4)  3168 (13.2)  3384 (14.1)  3392 (14.1)  3369 (14.0)  3492 (14.6)  3728 (15.5)  0 (0) | 9713 (14.9)  8990 (13.8)  9128 (14.0)  8937 (13.7)  9040 (13.9)  9497 (14.6)  9717 (14.9)  23 (0) | 0.018 |
| Public holiday  No  Yes | 23168 (96.6)  811 (3.4) | 62944 (96.8)  2101 (3.2) | 0.267 |
| Time of the call  Day (0800-1959)  Night (2000-0759)  NA | 15281 (63.7)  8647 (36.1)  51 (0.2) | 42667 (65.6)  22082 (33.9)  296 (0.5) | **<0.0001** |
| Working hours  No  Yes  NA | 13629 (56.8)  10299 (43.0)  51 (0.2) | 35872 (55.1)  28877 (44.4)  296 (0.5) | **<0.0001** |
|  |  |  |  |
| Bystander data |  |  |  |
| Type of bystander  Family  Health professional  Rescuer  Other  NA | 15838 (66.0)  2602 (10.9)  888 (3.7)  4617 (19.3)  34 (0.1) | 34431 (52.9)  11659 (17.9)  3057 (4.7)  10037 (15.4)  5861 (9.0) | **<0.0001** |
| tCPR  Yes  No  Missing value | 6070 (25.3)  7328 (30.6)  10581 (44.1) | 8131 (12.5)  16957 (26.1)  39957 (61.4) | **<0.0001** |
| CPR initiated  Yes  No | 12299 (51.3)  11680 (48.7) | 24516 (37.7)  40529 (62.3) | **<0.0001** |
|  |  |  |  |
| Patient data |  |  |  |
| Gender  Female  Male  NA | 7509 (31.3)  16466 (68.7)  4 (0) | 23071 (35.5)  41882 (64.4)  92 (0.1) | **<0.0001** |
| Age, mean (sd) | 67 (17) | 65 (20) | **<0.0001** |
| Age, by quartile  [18,56]  (56,69]  (69,81]  (81,108]  NA | 6157 (25.7)  6044 (25.2)  6209 (25.9)  5569 (23.2)  0 (0) | 17014 (26.2)  15296 (23.5)  14775 (22.7)  16151 (24.8)  1809 (2.8) | **<0.0001** |
| Cause of the CA  Medical cardiac  Medical non cardiac  Asphyxia  Traumatic  Drowning  Intoxication/Drug overdose  Electrocution | 15751 (65.7)  4615 (19.2)  1200 (5.0)  1852 (7.7)  390 (1.6)  160 (0.7)  11 (0) | 40473 (62.2)  10779 (16.6)  2707 (4.2)  5574 (8.6)  4258 (6.5)  1230 (1.9)  24 (0) | **<0.0001** |
| Known cardiovascular disease  Yes  Unknown | 10540 (44.0)  13439 (56.0) | 23853 (36.7)  41192 (63.3) | **<0.0001** |
| EDI (quintile)  1 – the least deprived  2  3  4  5 – the most deprived | 4055 (16.9)  3784 (15.8)  3553 (14.8)  4263 (17.8)  8324 (34.7) | 8346 (16.3)  8047 (15.7)  7222 (14.1)  9562 (18.7)  17968 (35.1) | **0.0017** |

Categorical variables are presented with number and percentage. Numerous variables are presented with mean and standard deviation.

CPR: Cardiopulmonary resuscitation, tCPR: CPR assisted by telephone

Appendix 2. Informations regarding OHCA process and patient outcomes, in accordance with the Utstein style

|  | Witnessed OHCA  N = 23,979 |
| --- | --- |
| BCLS data |  |
| Use of EAD before rescuers  Yes  No  NA | 1,750 (7.3)  15,928 (66.4)  6,301 (26.3) |
| BCLS team delay for arrival (min) | 12 (15) |
| No Flow (min) | 11 (13) |
| Use of EAD before MICU  Yes  No | 17,304 (72.2)  6,675 (27.8) |
| Chock delivered before MICU  Yes  No  NA | 4,685 (19.5)  11,309 (47.2)  7,985 (33.3) |
| ACLS data |  |
| ACLS team delay for arrival (min) | 22 (16) |
| First cardiac rhythm  ROSC  Asystole  Ventricular fibrillation or tachycardia  Pulseless electrical activity  NA | 1,359 (5.7)  17,781 (74.2)  2,337 (9.7)  1,504 (6.3)  998 (4.2) |
| Advance life support  Yes  No  NA | 18,155 (75.7)  5,818 (24.3)  6 (0) |
| Outcome |  |
| ROSC  Yes  No  NA | 6,744 (28.1)  17,222 (71.9)  13 (0) |
| ROSC delay (min) | 33 (19) |
| Pre hospital survival  Yes  No  NA | 6,196 (25.9)  17,779 (74.1)  4 (0) |
| Low Flow | 31 (20) |
| Survival at D30  Yes  No  NA | 1,868 (7.8)  22,103 (92.2)  8 (0) |
| Neurological outcome at D30  CPC 1  CPC 2  CPC 3  CPC 4  CPC 5  NA | 1,023 (54.5)  249 (13.3)  116 (6.2)  73 (3.9)  29 (1.5)  386 (20.6) |

Categorical variables are presented with number and percentage. Numerous variables are presented with mean and standard deviation.

OHCA: Out-of-hospital cardiac arrest, BCLS: Basic cardiac life support, EAD: External automatic defibrillator, MICU: mobile intensive care unit, ACLS: Advanced cardiac life support, ROSC: Return of spontaneous circulation, CPC: Cerebral performance
