# Supplementary material for: A national population-based study of patients, bystanders and contextual factors associated with resuscitation in witnessed cardiac arrest: insight from the french RéAC registry
Source: BMC Public Health. 2021 Dec 2;21:2202. doi: 10.1186/s12889-021-12269-4 (PMC8638114; doi:10.1186/s12889-021-12269-4)
Supplement: Supplementary file 2 — Additional file 2. [file 12889_2021_12269_MOESM2_ESM.docx]

**Supplementary data**

Table 1. Bivariate analysis between the deprivation level and the co variables.

|  | Total  N = 42999 | EDI | | | | | |
| --- | --- | --- | --- | --- | --- | --- | --- |
|  |  | 1  (the least deprived)  N=5961 | 2  N=5826 | 3  N=5320 | 4  N=6638 | 5  (the most deprived)  N=12234 | NA  N=7020 |
| Context data |  |  |  |  |  |  |  |
| Location  At home  Public place  Other  NA | 29937 (69.6)  8891 (20.7)  1139 (2.6)  3032 (7.1) | 4194 (70.4)  1149 (19.3)  129 (2.2)  489 (8.2) | 3974 (68.2)  1172 (20.1)  154 (2.6)  526 (9.0) | 3636 (68.3)  1098 (20.6)  154 (2.9)  432 (8.1) | 4518 (68.1)  1373 (20.7)  190 (2.9)  557 (8.4) | 8436 (69.0)  2536 (20.7)  315 (2.6)  947 (7.7) | 5179 (73.8)  1563 (22.3)  197 (2.8)  81 (1.2) |
| Witness status  Yes  NA | 42198 (98.1)  801 (1.9) | 5899 (99.0)  62 (1.0) | 5754 (98.8)  72 (1.2) | 5217 (98.1)  103 (1.9) | 6465 (97.4)  173 (2.6) | 11856 (96.9)  378 (3.1) | 7007 (99.8)  13 (0.2) |
| Presence of a rescue team  No  NA | Presence of rescue team  30832 (71.7)  12167 (28.3) | Presence of rescue team  4060 (68.1)  1901 (31.9) | Presence of rescue team  3786 (65.0)  2040 (35.0) | Presence of rescue team  3554 (66.8)  1766 (33.2) | Presence of rescue team  4269 (64.3)  2369 (35.7) | Presence of rescue team  8326 (68.1)  3908 (31.9) | Presence of rescue team  6837 (97.4)  183 (2.6) |
| Public holiday  No  Yes  NA | 41549 (96.6)  1450 (3.4)  0 (0.0) | 5752 (96.5)  209 (3.5)  0 (0.0) | 5640 (96.8)  186 (3.2)  0 (0.0) | 5144 (96.7)  176 (3.3)  0 (0.0) | 6423 (96.8)  215 (3.2)  0 (0.0) | 11795 (96.4)  439 (3.6)  0 (0.0) | 6795 (96.8)  225 (3.2)  0 (0.0) |
| Time of the call  Day (0800-1959)  Night (2000-0759)  NA | 27365 (63.6)  15445 (35.9)  189 (0.4) | 3856 (64.7)  2091 (35.1)  14 (0.2) | 3707 (63.6)  2103 (36.1)  16 (0.3) | 3370 (63.3)  1939 (36.4)  11 (0.2) | 4262 (64.2)  2328 (35.1)  48 (0.7) | 7715 (63.1)  4429 (36.2)  90 (0.7) | 4455 (63.5)  2555 (36.4)  10 (0.1) |
| Working hours  No  Yes  NA | 24324 (56.6)  18486 (43.0)  189 (0.4) | 3364 (56.4)  2583 (43.3)  14 (0.2) | 3334 (57.2)  2476 (42.5)  16 (0.3) | 3057 (57.5)  2252 (42.3)  11 (0.2) | 3706 (55.8)  2884 (43.4)  48 (0.7) | 6903 (56.4)  5241 (42.8)  90 (0.7) | 3960 (56.4)  3050 (43.4)  10 (0.1) |
|  |  |  |  |  |  |  |  |
| Bystander data |  |  |  |  |  |  |  |
| Type of bystander  Family  Health prof.  Rescuer  Other  NA | 27896 (64.9)  4737 (11.0)  1904 (4.4)  7651 (17.8)  811 (1.9) | 3974 (66.7)  667 (11.2)  273 (4.6)  984 (16.5)  63 (1.1) | 3821 (65.6)  683 (11.7)  283 (4.9)  960 (16.5)  79 (1.4) | 3460 (65.0)  568 (10.7)  248 (4.7)  933 (17.5)  111 (2.1) | 4249 (64.0)  756 (11.4)  315 (4.7)  1140 (17.2)  178 (2.7) | 7951 (65.0)  1242 (10.2)  522 (4.3)  2164 (17.7)  355 (2.9) | 4441 (63.3)  821 (11.7)  263 (3.7)  1470 (20.9)  25 (0.4) |
| tCPR  Yes  No  Missing value | 9065 (21.1)  11184 (26.0)  22750 (52.9) | 1423 (23.9)  1429 (24.0)  3109 (52.2) | 1230 (21.1)  1384 (23.8)  3212 (55.1) | 1153 (21.7)  1274 (23.9)  2893 (54.4) | 1405 (21.2)  1581 (23.8)  3652 (55.0) | 2304 (18.8)  2879 (23.5)  7051 (57.6) | 1550 (22.1)  2637 (37.6)  2833 (40.4) |
| CPR initiation  Yes  No | 21453 (49.9)  21546 (50.1) | 3229 (54.2)  2732 (45.8) | 3115 (53.5)  2711 (46.5) | 2763 (51.9)  2557 (48.1) | 3298 (49.7)  3340 (50.3) | 5606 (45.8)  6625 (54.2) | 3439 (49.0)  3581 (51.0) |
|  |  |  |  |  |  |  |  |
| Patient data |  |  |  |  |  |  |  |
| Gender  Female  Male  NA | 13576 (31.6)  29338 (68.2)  85 (0.2) | 1674 (28.1)  4281 (71.8)  6 (0.1) | 1778 (30.5)  4044 (69.4)  4 (0.1) | 1666 (31.3)  3639 (68.4)  15 (0.3) | 2082 (31.4)  4535 (68.3)  21 (0.3) | 4147 (33.9)  8052 (65.8)  35 (0.3) | 2229 (31.8)  4787 (68.2)  4 (0.1) |
| Age, by quartile  [18,56]  (56,69]  (69,81]  (81,108]  NA | 10933 (25.4)  10887 (25.3)  11016 (25.6)  10113 (23.5)  50 (0.1) | 1356 (22.7)  1547 (26.0)  1562 (26.2)  1488 (25.0)  8 (0.1) | 1360 (23.3)  1455 (25.0)  1577 (27.1)  1429 (24.5)  5 (0.1) | 1268 (23.8)  1349 (25.4)  1387 (26.1)  1308 (24.6)  8 (0.2) | 1628 (24.5)  1656 (24.9)  1651 (24.9)  1689 (25.4)  14 (0.2) | 3312 (27.1)  3145 (25.7)  3016 (24.7)  2749 (22.5)  12 (0.1) | 2009 (28.6)  1735 (24.7)  1823 (26.0)  1450 (20.7)  3 (0.0) |
| Cause of the CA  Med. cardiac  Med. non cardiac  Asphyxia  Traumatic  Drowning  Intox./Drug OD  Electrocution  NA | 27931 (65.0)  8138 (18.9)  2133 (5.0)  3780 (8.8)  693 (1.6)  308 (0.7)  16 (0)  0 (0) | 3943 (66.1)  1082 (18.2)  255 (4.3)  548 (9.2)  100 (1.7)  33 (0.6)  0 (0)  0 (0) | 3819 (65.6)  1075 (18.5)  307 (5.3)  489 (8.4)  101 (1.7)  32 (0.5)  3 (0.1)  0 (0) | 3496 (65.7)  966 (18.2)  280 (5.3)  470 (8.8)  77 (1.4)  30 (0.6)  1 (0.0)  0 (0) | 4296 (64.7)  1226 (18.5)  342 (5.2)  628 (9.5)  95 (1.4)  44 (0.7)  7 (0.1)  0 (0) | 7910 (64.7)  2317 (18.9)  621 (5.1)  1122 (9.2)  156 (1.3)  104 (0.9)  4 (0)  0 (0) | 4467 (63.6)  1472 (21.0)  328 (4.7)  523 (7.5)  164 (2.3)  65 (0.9)  1 (0.0)  0 (0) |
| Cardiovasc. disease  Unknown  Yes | 25451 (59.2)  17548 (48.0) | 3506 (58.8)  2455 (41.2) | 3472 (59.6)  2354 (40.4) | 3210 (60.3)  2110 (39.7) | 3974 (59.9)  2664 (40.1) | 7304 (59.7)  4930 (40.3) | 3985 (56.8)  3035 (43.2) |

Categorical variables are presented with number and percentage.

CA: Cardiac arrest, CPR: Cardiopulmonary resuscitation, tCPR: CPR assisted by telephone, EDI: European Deprivation Index, OD: Overdose

Table 2. Multivariable model for initiation of bystander cardiopulmonary resuscitation with imputated data.

|  | OR (IC 95%) | p value |
| --- | --- | --- |
| EDI (quintile)  1 – the least deprived  2  3  4  5 - the most deprived | Ref  0.96 (0.90-1.02)  0.94 (0.88-1.00)  0.83 (0.77-0.88)  0.72 (0.72-0.81) | 0.170  **0.042**  **<0.001**  **<0.001** |
| Location  At home  Public place  Other | Ref  2.26 (2.14-2.40)  1.96 (1.77-2.17) | **<0.001**  **<0.001** |
| Working hours  No  Yes | Ref  1.02 (0.99-1.06) | 0.227 |
| Type of bystander  Family  Health prof.  Rescuer  Other | Ref  10.35 (9.84-10.87)  19.09 (17.33-21.04)  0.99 (0.94-1.04) | **<0.001**  **<0.001**  0.629 |
| tCPR  Yes  No  Missing value | Ref  0.08 (0.07-0.08)  0.12 (0.11-0.12) | **<0.001**  **<0.001** |
| Gender  Female  Male | Ref  1.06 (1.02-1.10) | **0.001** |
| Age, by quartile  [18,57]  (57,70]  (70,82]  (82,108] | Ref  0.85 (0.81-0.89)  0.72 (0.68-0.75)  0.53 (0.51-0.56) | **<0.001**  **<0.001**  **<0.001** |
| Cause of the CA  Med. cardiac  Med. non cardiac  Asphyxia  Traumatic  Drowning  Intox./Drug overdose  Electrocution | Ref  1.00 (0.96-1.05)  1.52 (1.41-1.65)  0.36 (0.34-0.39)  0.68 (0.63-0.73)  0.60 (0.52-0.69)  1.76 (0.80-3.84) | 0.836  **<0.001**  **<0.001**  **<0.001**  **<0.001**  0.160 |
| Cardiovasc. disease  Unknown  Yes | Ref  1.20 (1.15-1.24) | **<0.001** |

The multivariable analysis was performed on 42,999 patients without missing data.

CA: Cardiac arrest, CPR: Cardiopulmonary resuscitation, tCPR: CPR assisted by telephone, EDI: European Deprivation Index, OR: odds ratio
